# Determination of nest occupation and breeding effect of the white stork by human-mediated landscape in Western Poland

**DOI:** 10.1007/s11356-019-06639-0

**Published:** 2019-12-11

**Authors:** Joanna T. Bialas, Łukasz Dylewski, Marcin Tobolka

**Affiliations:** 1 Institute of Zoology, Poznań University of Life Sciences, Wojska Polskiego 71C, 60-625 Poznań, Poland; 2 Institute of Dendrology, Polish Academy of Sciences, Parkowa 5, 62-035 Kórnik, Poland

**Keywords:** Breeding effect, Nest-site selection, Habitat selection, Nest occupation, Landfills, *Ciconia ciconia*

## Abstract

Choosing an appropriate nest site is essential for successful breeding. Changes in land use cause populations of many species to decline although some species adapt to anthropogenic changes. The white stork *Ciconia ciconia* commonly uses artificial nest sites. Recently, white storks from Western Europe have been using landfills as feeding sites; the beginnings of this process are being observed in Central-Eastern Europe. The study aimed to determine factors influencing the probability of nest occupation and breeding effect in a Central-Eastern European population of white storks. We used long-term data from Western Poland on breeding effect, nest occupation, the structure supporting the nest, the proximity of the nearest landfills, landfill area, and land cover. The probability of nest occupation was significantly dependent on habitat quality (based on the share of the preferred type of land cover), the structure supporting the nest, and landfill proximity within a specific year. The breeding effect was influenced by habitat quality and nesting structure. We demonstrate that the type of nesting structure is an important factor influencing both the probability of nest reoccupation and breeding effect. However, the significance of landfills appears to be growing, and in recent years, storks prefer occupying nests closer to landfills, which may have significant consequences for the population of the white stork.

**Electronic supplementary material:**

The online version of this article (10.1007/s11356-019-06639-0) contains supplementary material, which is available to authorized users.

## Introduction

For animals, choosing a breeding site is of great significance for successful reproduction (Horn 1968; Birkhead 1977; Partridge 1988). Oviparous animals, e.g. birds, spend several weeks or months in one location to raise their offspring. Their decisions concerning when and where to breed have enormous consequences for breeding success and fitness (Perrins 1970; Partridge 1988). The quality of such a territory is a key factor which reliably explains occupation over time (Janiszewski et al. 2013). Territory quality may be reflected in the share of preferred habitats in the immediate surroundings of the nest and may affect reproductive output (Reijnen and Foppen 1994; Lambrechts et al. 2004; Tobolka et al. 2012).

### The structure supporting the nest

Nest-site selection is vital mainly because of nest predation (Martin 1993). Many aspects of nest-supporting structures and location are important and may influence the breeding success of solitary nesting birds: nesting shrub species (Tryjanowski et al. 2000), tree species, height, and diameter (Zawadzki and Zawadzka 2017). For some bird species, the type of nest supporting structure explains not only breeding effect but also reoccupation rate (Tryjanowski et al. 2009; Tobolka et al. 2013; Janiszewski et al. 2015). Anthropogenic changes in the environment generally have a negative influence on the availability of natural nesting sites (Yasué and Dearden 2006); however, for some bird species, changes in the environment lead to the provision of new sites (Mainwaring 2015). The white stork *Ciconia ciconia* is a bird widely known for its association with anthropogenic habitats, as it nests in close proximity to human settlements (Bairlein 1991). White storks often reuse nests built in previous years, and despite high site fidelity of pairs (Vergara et al. 2006; Barbraud et al. 2008), storks observed in one year are not necessarily the same birds as in previous year. As a behaviourally plastic species, the white stork can change its nesting behaviour in response to the availability of nesting sites (Tobolka et al. 2013) and nesting material (Jagiello et al. 2018). In this species, natural nesting structures such as trees have slowly given way to roofs of human settlements and high chimneys, and currently to electrical pylons, which are now the most common nest sites for the white stork in Central-Eastern Europe (Tryjanowski et al. 2009; Janiszewski et al. 2015; Vaitkuvienė and Dagys 2015). However, this change in the selection of structures supporting nests by the white stork has not affected the breeding effect or nest reoccupation rate pattern (Tryjanowski et al. 2009; Janiszewski et al. 2015).

### Habitat loss and anthropogenic food sources

The quality of such a territory is a key factor which reliably explains occupation over time (Janiszewski et al. 2013). This quality may be reflected in the share of preferred habitats in the immediate surroundings of the nest and may affect reproductive output (Reijnen and Foppen 1994; Lambrechts et al. 2004; Tobolka et al. 2012). Birds which inhabit human-changed environments, e.g. agricultural lands, rely on human management and thus may suffer from further unfavourable changes to habitats, e.g. intensification of agricultural practices (Chamberlain et al. 2000; Donald et al. 2001, 2006). However, progressive anthropogenic changes to the environment and new food sources induce wild animals to change their foraging behaviour, e.g. to use artificial food sources (Plaza and Lambertucci 2017). This has a significant influence on the biology and ecology of animals, and leads to consequences for fitness, reproductive success, demographic changes in the whole population, risk of pathogen infection, toxin ingestion (reviewed in Plaza and Lambertucci 2017), and even changes in migratory behaviour (Gilbert et al. 2016). The white stork is included among species that suffer from habitat loss caused by agricultural intensification (Bairlein 1991)*.* Its primary foraging sites are meadows, river valleys, wetlands, and pastures (e.g. Schulz 1998; Tobolka et al. 2012, 2013), but it is an opportunist in terms of food, i.e. it uses the most easily acquired and the most abundant food (Kosicki et al. 2006). Opportunistic foraging birds may exhibit a lower level of neophobia towards new food items (Cambefort 1981) and/or foraging sites. In Western Europe and North Africa, the use by white storks of landfills as foraging sites is frequently observed (Rey 2009; Djerdali et al. 2016a). As the energetic costs of breeding are very high, food abundance plays a crucial role in limiting breeding success (Martin 1987). Hence, foraging at landfills which provide stable and constant access to food of high energy content has raised the Western European population’s productivity level (Tortosa et al. 2003; Djerdali et al. 2008a, 2016b) and ultimately its size (Djerdali et al. 2016a) following a tremendous collapse in the past (Bairlein 1991). Landfills, as a certain anthropogenic source of food, have also affected the migration strategy of white storks (Gilbert et al. 2016; Massemin-Challet et al. 2006). Western migratory population of the white stork originally migrates via Gibraltar to Western Africa and to Sahel Zone (Flack et al. 2016). Currently, a significant fraction of the breeding population has become sedentary (Sanz-Aguilar et al. 2015), and other groups stop the migration in Europe or North Africa. For example, in the German population of white storks, over 80% out of 169 juveniles equipped with transmitters stayed in Europe or North Africa, which increased their survival probability (Cheng et al. 2019). Many of them use landfills as stopovers sites and foraging grounds (Arizaga et al. 2018). However, in Central-Eastern Europe, where the eastern migratory white stork population occurs, the use of landfills is still a rather novel behaviour (Kruszyk and Ciach 2010); thus, to date, no studies have been conducted aimed at assessing the effect of landfill proximity on the breeding ecology of the white stork. Nevertheless, such behaviour seems to be growing more frequent, especially during migration to wintering grounds (Ciach and Kruszyk 2010). Moreover, for long-lived birds, the experience gathered in a previous season may be used in subsequent breeding attempts (Ciach and Kruszyk 2010). However, there is still a lack of knowledge regarding the scale of the phenomena in Central-Eastern Europe, as well as the consequences of foraging at landfills for this population.

### Study aims

As mentioned above, the proximity of a landfill increases the breeding success, breeding effect, and changes the migratory behaviour of white storks, as is visible in the recovering population in Western Europe. However, no studies have accounted for two other significant factors, nesting structure and the surrounding area, or natural/semi-natural foraging grounds. Although the effect of land cover on the ecology of the white stork has been thoroughly studied in Central-Eastern Europe (Tobolka et al. 2012; Janiszewski et al. 2013), the effect of landfills has never been part of this research. What is more, the effect of structure supporting the nest on the ecology, on the basis of previous studies, is still unclear. The aim of this study was to determine which of these factors are main drivers of the reoccupation rate and the productivity of the white stork.

## Methods

### Study area

The study was conducted in 2007–2017 in western Poland near the town of Leszno (51° 51′ N, 16° 35′ E) within an area, dominated by agricultural land, of 4154 km^2^. This area consists mainly of arable fields (54%) interspersed with meadows (7%), pastures (less than 1%), human settlements (10%), forests (17%), and others like set-asides, orchards, or industrial areas (all together up to 11%) (Tobolka et al. 2013). The white stork is mostly a solitary breeder in western Poland, but small aggregations of up to five pairs are also observed, mainly in small river valleys. The population density ranged from 5.24 to 6.76 pairs/100 km^2^ (Tobolka et al. 2013, 2015, 2018).

### Data collection

In the years 2007–2017, we collected data on the breeding effect (expressed as a number of fledglings) and nest occupancy of a total of 2768 pairs (a yearly average of 278, range 246–331) from 4313 nests (yearly average 359, range 366–407). Each year, we visited each nest directly at least twice: the first visit at the beginning of the breeding season in April, in order to detect pairs which had occupied nests, the second in the first half of July, regardless of the actual phenological stage of broods. Then we recorded the number of fledglings standing on the nest and considered able to fly, which we defined here as the breeding effect, according to a standard method used in the monitoring of the white stork (Profus 2006). Both visits covered the entire study area and each detected nest. When necessary, we conducted additional visits, mainly to late broods, when the chicks were too small in July for us to assume their ability to fly. We supplemented all uncertain records by interviews with householders living near the nest; if they did not clarify the breeding status of the nest, we excluded the brood from further analyses (Tobolka et al. 2015). As occupied nests (1), we coded nests built in previous years and newly built nests with a breeding pair (based on criteria proposed by Profus (2006)). Nests with non-breeding pairs, visited irregularly, or without any visitors were coded as unoccupied (0). We obtained data on the number of breeding pairs (HPa), pairs with fledglings (HPm, with exact numbers of offspring), and pairs which failed to reproduce (HPo). We also noted nesting structure type of each nest. We divided nesting structures into categories: pylons (averagely 291 a year), trees (26), roofs (41), chimney (72), and other man-made structures such as fire sirens or hunting towers (8). We did not collect data on all the places suitable to hold the nest but only on structures that held nests (built in previous years or new) in each year.

We obtained data on landfill locations and areas in each year of the study from the Provincial Environmental Protection Inspectorates of the Greater Poland, Lower Silesia, and Lubusz Voivodeships. As some of the landfills had been closed and additional sites opened elsewhere during the years of our investigation, the distance from a given nest to the nearest landfill sometimes differed from year to year. Therefore, we calculated this value separately for each nest for each individual year of the study.

### Spatial data analysis

For all spatial analysis, we used QGIS 2.18.13 open-source software. Analyses consisted of calculating the distance from each nest to the nearest landfill and obtaining information about the land cover within a radius of 2 km of each nest using CORINE Land Cover (2006, 2012). Because white storks show a preference for a particular type of habitat, i.e. meadows, pastures, and wetlands avoiding great complex of forests (e.g. Tobolka et al. 2012, Janiszewski et al. 2013, Radović et al. 2014), we used land cover data as a proxy for habitat quality in this study. Data on the land cover, to some extent, should reflect the data that storks obtain while searching for suitable habitat. Basing on this data, they establish territories and we can assume that the patches of preferable quality should cover as much of a home range as possible. As we wanted to avoid assigning quality of the patch arbitrarily, we decided to use only the coverage of the patches in the whole territory to be able to show which of these are significant in the process of choosing nest-site and breeding effect. The home range was established on the basis of previous studies of the white stork (Nowakowski 2013; Zurell et al. 2018), and its radius was 2 km. To keep up with changes in land cover over time and closing and opening of landfills, spatial analysis for each nest was conducted separately for each year.

We used the CORINE Land Cover (European Commission 1993) spatial database, which provides a pan-European inventory of biophysical land-cover classes. The database, which uses 44 class names at the third (highest) level of detail, is a key resource for integrated environmental assessments. We used third-level physical and physiognomic entities. The CORINE Land Cover map was created from remotely sensed image data from the SPOT and IRS satellite programmes. The database has been validated; the official classification accuracy of CORINE has been reported at 87% (European Environmental Agency). The data used for the analysis was considered appropriate for the spatial and temporal scale of the presented investigation and had been used previously in studies of the white stork (Radović and Tepić 2009; Tobolka et al. 2012).

We used vector data available at the Chief Inspectorate of Environmental Protection website (http://www.eea.europa.eu/data-and-maps). The datasets used in this work represent land cover in 2006 for nests existing from 2007–2011 and 2012 for nests existing in 2012–2017. We used the processing plug-in for QGIS to analyse the share of 20 land-cover classes in the 2-km-radius buffers created around each nest. We used the following classes: continuous urban fabric (> 80% of the land surface is covered by impermeable features like buildings, roads, and artificially surfaced areas); discontinuous urban fabric (impermeable features range from 30 to 80% land coverage); industrial or commercial units; mineral extraction sites; construction sites; green urban areas; sport and leisure facilities; non-irrigated arable land; fruit trees and berry plantations; pastures; complex cultivation patterns; land principally occupied by agriculture, with significant areas of natural vegetation; broad-leaved forest; coniferous forest; mixed forest; transitional woodland-shrub; sparsely vegetated areas; inland marshes; water courses; water bodies. Then we assembled these classes into seven groups appropriate for studied species to make data easier to analyse and present*:* areas greatly altered by humans (including continuous urban fabric, discontinuous urban fabric, industrial or commercial units, mineral extraction sites, construction sites, green urban areas, and sport and leisure facilities); non-irrigated arable land; other agricultural land (fruit trees and berry plantations; complex cultivation patterns; land principally occupied by agriculture, with significant areas of natural vegetation); pastures and meadows; forests (broad-leaved forest, coniferous forest, mixed forest, transitional woodland-shrub); inland marshes; inland waters (water courses, water bodies).

### Statistical analysis

To determine which factors influence the probability of nest occupation and white stork breeding effect (output), we used generalised linear mixed models (GLMMs) with restricted maximum-likelihood (REML) estimator implemented. As the probability of nest occupation, we took binary data on nest occupation in each year (occupied or not, only for existing nests, not the structures possible to hold nests). In both models, we used nest ID and year as random factors. The first model (GLMM_1) included the probability of nest occupation as a dependent variable with a binomial error structure and logit link function. The second model (GLMM_2) included breeding effect as a dependent variable with Gaussian error structure and identity link function. In the structures of both models, we included nesting structure (nest_str), distance to landfill (dist_land), area of landfill (land), share of areas greatly altered by humans (human), non-irrigated arable land (arable), other agricultural land (agri_land), pastures and meadows (meadow), and forests (forest). In all analyses, distance to landfill (+ 1) was natural log-transformed. In both models, the full model included the following interaction: years and distance to landfill (year × dist_land), nesting structure and distance to landfill (nest_str × dist_land*)*, nesting structure and area of landfill (nest_str × land), nesting structure and share of areas greatly altered by humans (nest_str × human), nesting structure and non-irrigated arable land (nest_str × arable), nesting structure and other agricultural land (nest_str × agri_land), nesting structure and pastures and meadows(nest_str × meadow). We also included a quadratic term for non-irrigated arable land to allow for a non-linear relationship in both models, as supported by improvement of the model AICc score (AICc = − 2.94; AICc = − 2.77, respectively, for GLMM_1 and GLMM_2) as calculated with maximum-likelihood estimation. To avoid multicollinearity, we excluded three variables (shares of forests, inland marshes, and inland waters) from both models. Multicollinearity in the remaining explanatory variables in both models was not excessive (VIF < 2).

We employed the information-theoretic approach (Burnham and Anderson 2002) to identify the most parsimonious models explaining variation in all dependent variables. Based on the full model, in each analysis, we constructed a set of candidate models as calculated with maximum-likelihood (ML) estimation that included different combinations of the predictors. For model selection, we used the Akaike Information Criterion, adjusted for small sample sizes (AICc). We used the best models with the lowest AICc values. The results of the model selection procedure are presented in Table S1 and Table S2. The final model validation was checked using diagnostic plots in both cases.

When significant results for categorical variable (nesting structure) were obtained from the GLMMs, we carried out pairwise tests using multiple comparisons with a Šidák correction in the first model and a Bonferroni correction in the second. We used diagnostic plots for final validations of the models. All analyses were carried out in R 3.3.2 (R Core Developmental Team, 2016). GLMMs were carried out using the lme4 package (Bates et al. 2015). Model selection was accomplished using the MuMIn package (Bartoń 2018). The pairwise tests were carried out using the lsmeans package (Lenth 2016), the data visualisation using the ggplot2 package (Wickham 2016).

## Results

**Fig. 1:**
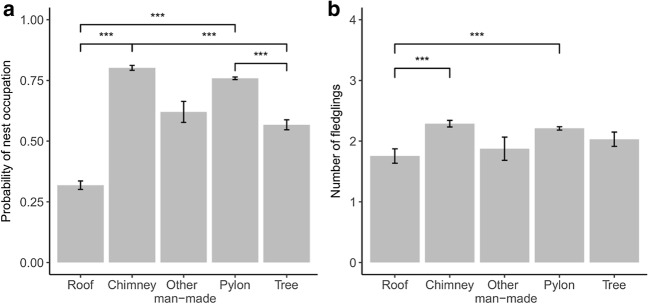
The relationship between the probability of nest occupation (**a**) and number of fledglings (**b**), and type of nesting structure

**Fig. 2:**
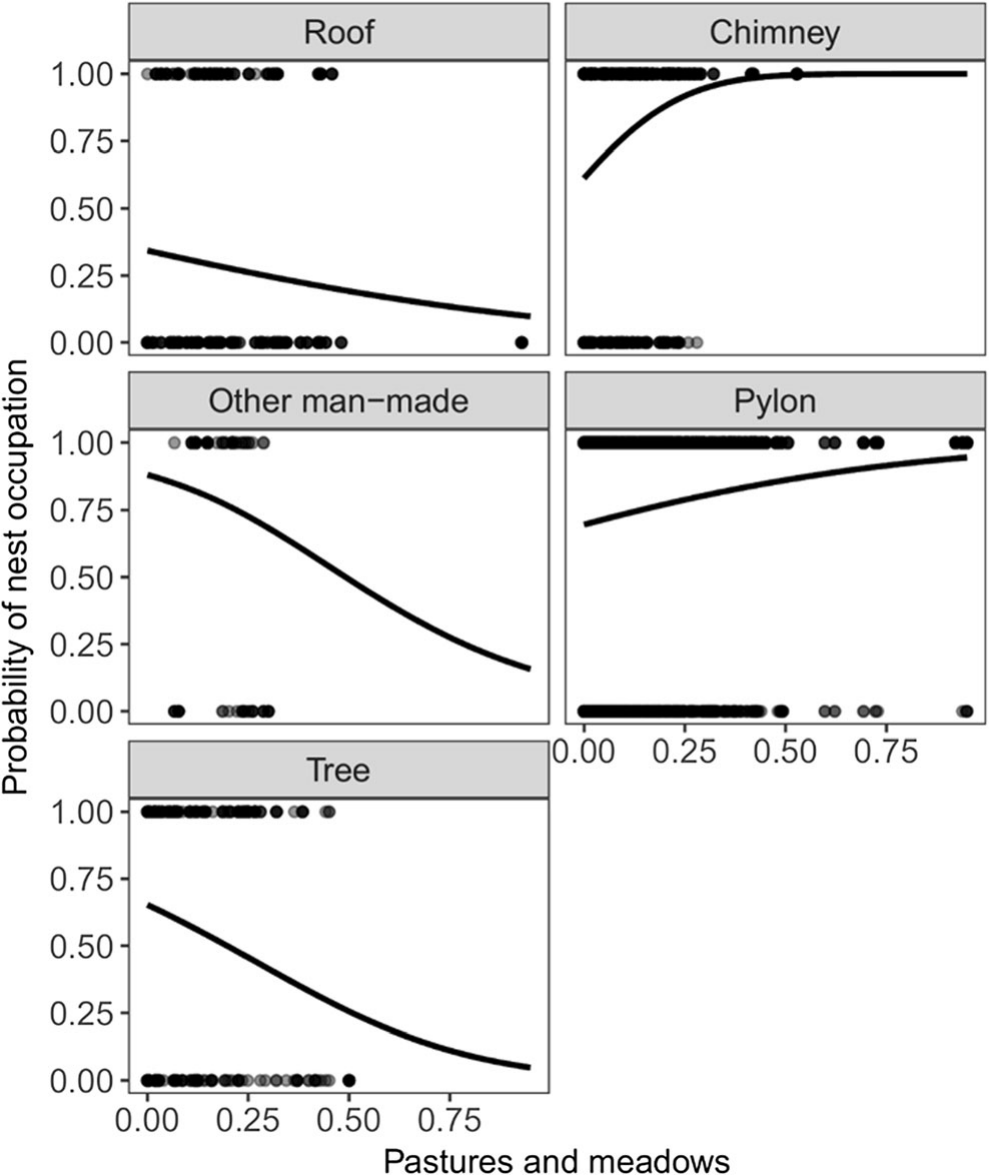
The relationship between the probability of nest occupation and proximity of pastures and meadows on different nesting structures

**Fig. 3:**
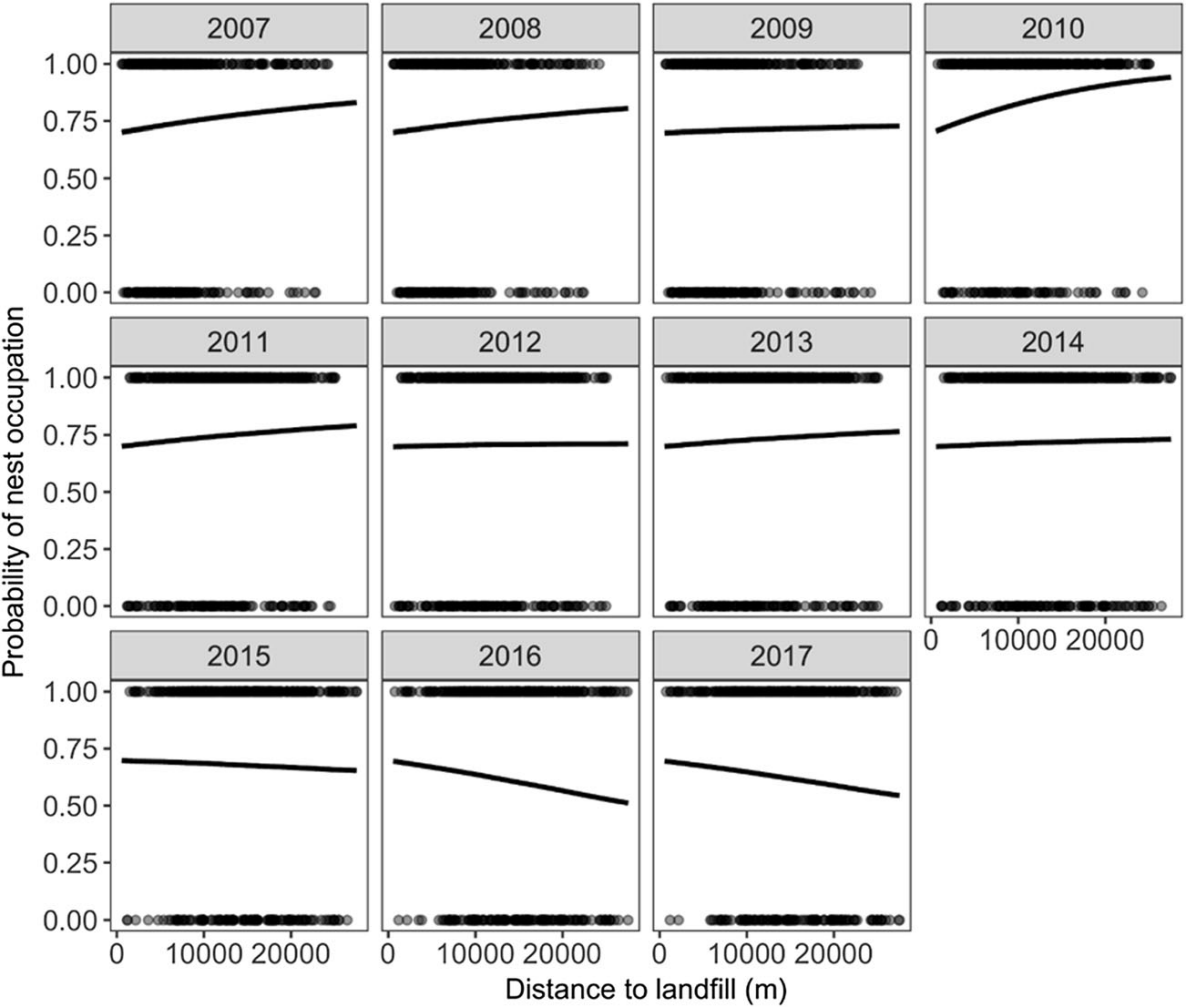
The relationship between the probability of nest occupation and distance to landfill in subsequent years

Based on the best model for first GLMM analysis, we found differences in probability of nest occupation between nesting structures (Table 1, Fig. 1). The highest probability of nest occupation was observed on pylons and chimneys, and it was significantly higher than the probability of nesting on roofs and trees. We also found that the cover of areas greatly altered by humans, other agricultural land, and pastures and meadows were positively correlated with probability of nest occupation (Table 1). The probability of nest occupation was also related to different nesting structures within pastures and meadows, highly human-changed areas, and landfills areas (Table 1, Fig. 2). We also found that the interaction between years and distance to landfill significantly affected the probability of nest occupation (Table 1, Fig. 3). Occupation probability on chimneys and pylons grows with the growing cover of pastures and meadows, but in the case of roofs, trees, and other man-made structures, the probability decreases with decreasing cover of pastures and meadows. In the last 2 years, we observed storks to prefer nesting closer to landfills. Probability of nest occupation is also significantly affected by the interaction of landfill area and nesting structure.

**Table 1:** The GLMMs’ with binomial error structure and logit link function, describing the relationship between the probability of nest occupation in white storks with land cover, nest location, and distance to the nearest landfill

Variable	Wald	df	*P*
Nest_str	*66.27*	*4*	*< 0.0001*
Dist_land	0.21	1	0.643
Human	*18.07*	*1*	*< 0.0001*
Land	0.64	1	0.424
Arable	*6.38*	*1*	*0.012*
Arable2	*4.51*	*1*	*0.034*
Agri_land	0.40	1	0.526
Meadow	*4.24*	*1*	*0.039*
Year × dist_land	*149.20*	*10*	*< 0.0001*
Nest_str × dist_land	7.13	4	0.129
Nest_str × human	*16.66*	*4*	*0.002*
Nest_str × land	*11.40*	*4*	*0.022*
Nest_str × arable	8.97	4	0.062
Nest_str × agri_land	4.49	4	0.343
Nest_str × meadow	*12.76*	*4*	*0.013*

*nest_str* nesting structure, *dist_land* distance to landfill, *land* area of landfill, *human* share of areas greatly altered by humans, *arable* non-irrigated arable land, *agri_land* other agricultural land, *meadow* pastures and meadows, (×) interactions between these variables

In italics are marked significant predictors

**Fig. 4:**
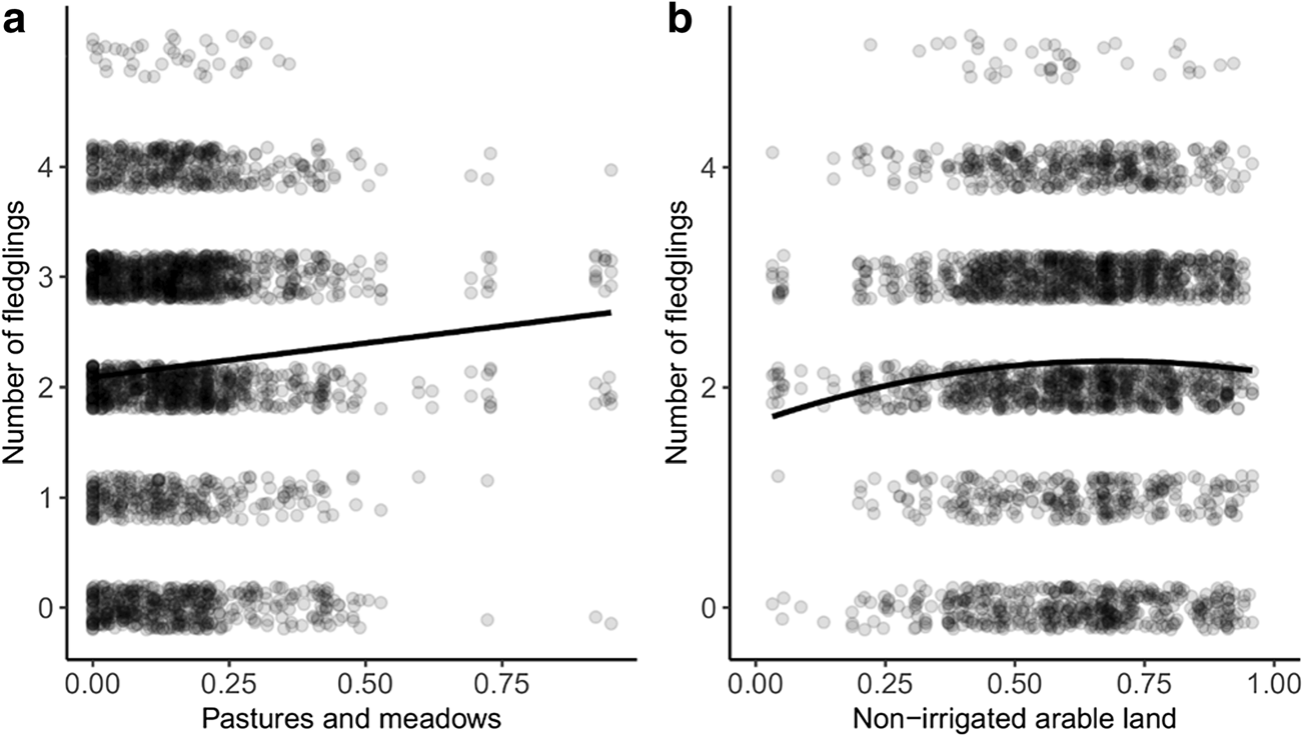
The relationship between **a** breeding effect (number of fledglings) and **b** land cover types

Based on the best model for the second GLMM analysis, we found that the effects of nesting structure and cover of arable land were significant predictors of number of fledglings raised by a pair (breeding effect) (Table 2). Storks had significantly higher breeding effect on chimneys and pylons than on roofs. Cover of pasture and meadows had a positive effect on breeding effect (Fig. 4). However, the cover of arable land showed a non-linear effect—positive when share of arable land is up to ca 70% and negative over this value (Fig. 4).

**Table 2:** The GLMMs’ with Gaussian error structure describing the relationship between breeding effect of white storks with land cover, nest location, and distance to the nearest landfill

Variable	*F*	df	*P*
Nest_str	*23.46*	*4*	*< 0.0001*
Dist_land	0.07	1	0.788
Human	0.56	1	0.456
Arable	*5.37*	*1*	*0.020*
Arable^2^	*4.16*	*1*	*0.041*
Agri_land	0.21	1	0.648
Meadow	*5.99*	*1*	*0.014*
Year × dist_land	11.26	10	0.338
Nest_str × dist_land	6.38	4	0.172

*nest_str* nesting structure, *dist_land* distance to landfill, *human* share of areas greatly altered by humans, *arable* non-irrigated arable land, *agri_land* other agricultural land, *meadow* pastures and meadows, *year × dist_land* interaction between years and distance to landfill, *nest_str × dist_land* interaction between nest structure and distance to landfill

In italics are marked significant predictors

## Discussion

We show that the probability of nest occupation depends on the share of particular habitats in the vicinity of the nest, structure supporting the nest, and distance to the nearest landfill in connection with a particular year, and that breeding effect is related to habitat composition and nesting structure.

### Habitat selection

Our results show that land cover, which represents habitat quality, is crucial in nest-site selection, consistent with previous findings (Janiszewski et al. 2013), and that the most important habitats for white storks are pastures and meadows, agricultural areas, and areas greatly altered by humans (including urban fabric, industrial, commercial and transport units, mine, dump, and construction sites, and artificial, non-agricultural vegetated areas). However, the relationship between the probability of nest occupation and the share of non-irrigated arable land in the buffer zone around white stork nests was not linear. These habitats are necessary for the storks but when they cover a certain percentage (ca 70%, ESM Fig. S1) the probability of nest occupation does not increase significantly. The relationship between nest occupation and share of pastures and meadows and other agricultural areas was linear. We also found a relationship between habitat composition and the number of fledged chicks. Again, in the case of pastures and meadows, this relationship is linear and positive, whereas in the case of non-irrigated arable land, it is non-linear; this kind of land cover positively influences productivity until its share reaches a certain point (ca 70%). This finding is consistent with the fact that white storks breed in open habitats which have been changed by agro-technical treatments and which are located near human settlements (Profus 2006). White storks are known to use meadows more frequently exclusively in the main part of the breeding season (during incubation and chick rearing), whereas at the begging and the end of the breeding season, they forage in other agricultural areas just as often as in meadows (Rachel 2006). Another study shows that territories associated with large river valleys, as well as those in close proximity to wetlands, are strongly preferred; moreover, brood reduction measured with the chicks intentionally expelled from the nest by the parents in these territories is lowest (Nowakowski 2013; Janiszewski et al. 2014). However, using data on land cover enabled us to obtain more detailed information on habitat selection by white storks. Methods previously used to obtain data on land cover, in the light of our results, appears insufficient. The Corine Land Cover Database was used in a study of the habitat selection of white storks in Croatia (Radović and Tepić 2009), where distance to the nearest grasslands, distances to the three nearest agricultural mosaic habitats, and standardised proportions of forests in terms of area were important factors. Studies in Romania showed a relationship between breeding effect and altitude, number of river sectors, and distance to the nearest empty nest (Fasolă-Mătăsaru et al. 2018). Habitat selection, as well as its connection with breeding success, has been studied in many other farmland species under pressure from intensified farming. Other studies using the CLC Database showed that other bird species prefer extensively used farmland, i.e. the occurrence of semi-natural habitats increases the probability of breeding for great grey shrike *Lanius excubitor* or red-backed shrikes *Lanius collurio*, as well as positively affecting their breeding success (Kuczyński et al. 2010; Morelli 2012).

### Structures supporting nests

While the relationships between the probability of nest occupation and breeding effects and components of land cover have been discussed in several papers on the white stork (Radović and Tepić 2009; Janiszewski et al. 2013; Radović et al. 2014) and other birds (Kuczyński et al. 2010; Morelli 2012), studies evaluating the role of nesting structure in the occupation of territory are scarce. Here, we show that nesting structure is crucial for habitat selection, at least for the white stork, which contradicts two earlier papers concerning this species (Tryjanowski et al. 2009; Janiszewski et al. 2015). We found that storks tend to occupy nests located on chimneys and pylons rather than trees and roofs. Moreover, we found that breeding effect was also higher on chimneys and pylons. Previous studies showed that the transition to electrical pylons is probably driven by the lack of traditional nesting sites, as the use of pylons was most frequent in the best-quality habitats where competition for nest sites is the highest (Janiszewski et al. 2015). The fitness benefits of nesting on traditional structures were found in poor-quality habitats (Janiszewski et al. 2015), or not at all (Tryjanowski et al. 2009). A recent study from Western Europe showed that the use of pylons is connected with distance from feeding areas (Moreira et al. 2018) and is not driven by the lack of other nesting sites. Moreover, nesting on pylons may carry a risk of electrocution and it provides no protection from rain or overheating (D’Amico et al. 2018). The differences between these studies and ours may arise from the differences in abundancy of nesting structures in study sites. Our results show also that there is relationship between nesting structure and habitat quality in the case of occupation probability. Occupation probability on chimneys and pylons is higher on pastures and meadows, but in the case of roofs, trees, and other man-made structures, the probability is higher when the cover pastures and meadows are smaller. This suggests that the nesting structure itself does not impact the occupation probability. Differences in breeding effect and hence probability of occupation between different nesting structures may arise due to higher food abundancy in territories where nests are located on pylons but we did not find an interaction between habitat composition and nesting structure type. Another explanation is simply that pylons are just more abundant than roofs or trees appropriate to hold the nest and hence the sample from the pylons was much bigger. This may have caused results to be biased towards more frequent occupation of pylons and chimneys than other structures. However, many of the nests situated on roofs or trees were abandoned by storks, and new nests were built on pylons nearby. Unfortunately, the process of transition from traditional nesting structures to pylons has happened since last few decades; thus, it may be difficult to follow through. One of possible explanations of the transition to pylons is that differences in breeding success in the past has arisen because different levels of the risk of nest depredation. Only a few species represent potential predators of white stork chicks: martens *Martes* sp., the white-tailed eagle *Haliaeetus albicilla*, and the golden eagle *Aquila chrysaetos* (Jakubiec 1991; Jakubiec and Peterson 2005; Tobolka 2014). Chimneys and electrical pylons appear to be less accessible for land-based predators such as martens in comparison with trees or roofs (Jakubiec 1991), but nowadays, predation rate in white stork is too small to conclude that it has any impact on choosing nesting structure type; weather conditions play the main role in brood reduction (Tobolka et al. 2015). On the other hand, neither this nor previous studies contain data on the recruitment rate of individuals from nests built on different nesting structures, which may reveal whether the differences in number of fledglings related to nesting structure are also apparent in the number of juveniles which return as breeders (as a measure of fitness). Study based on ring recoveries would yield this important information on the recruitment rate of individuals and help to assess the role of nesting structure in the population trends.

### Anthropogenic food sources

Opposite to Western European population of the white stork which profit from foraging on landfills on the brood level (Tortosa et al. 2003), on the population level (Blanco 2006; Djerdali et al. 2016b), and also during migration (Massemin-Challet et al. 2006; Rotics et al. 2017; Arizaga et al. 2018), however sometimes suffer (de la Casa-Resino et al. 2014, 2015), the consequences of feeding at landfills for Central-Eastern European white stork populations are unknown. Our results show that proximity to a landfill determines the probability of nest occupation, but strictly in connection with a particular year. Since 2015, the probability of nest occupation becomes higher when closer to landfill. This suggests that in years 2015–2017, the use of anthropogenic food from landfills became important. Avoidance of landfills in previous years can be explained by the poor habitats in which landfills are located, e.g., in closed gravel pits, which, in Poland, are usually situated near coniferous forests. In the year 2010, storks avoided close proximity to landfills the most which is probably connected to weather conditions. In 2010, which was extremely humid, hence prey was abundant, we observed unusually many new nests been built, and all of them where located in close proximity to meadows (Tobolka et al. 2013). In Algeria, the proximity of landfills was a significant predictor for breeding effect, except the one very dry year of the study (Djerdali et al. 2016b), which contradicts our results and may be due to differences in food composition between different population of white stork under different climate (compare, e.g. Kosicki et al. 2006 and Chenchouni 2016). Nevertheless, a trend to nest closer to landfills is visible, also in population of storks from Central-Eastern Europe, where the foraging on landfills is a developing phenomenon (Kruszyk and Ciach 2010). The graphical visualisation based on our model GLMM1 showed that there is a change in the relationship between probability of nest occupation and the distance to landfills. Although the trend for the whole population is not significant, the differences between study years are significant, i.e. in last years, the probability of nesting close to landfill is increasing, which indicates that the process may develop in the same direction as in Western Europe (Fig. 3). In recent years, on this particular study area, we have even observed the construction of new nests in villages close to landfills (authors’ observations), and this process is also beginning in Eastern Poland (I. Kaługa, K. Pawlukojć personal observations). This process has also been observed in Western European populations. Since the mid-1980s, when a new method of rubbish treatment was introduced (open air landfills where garbage were collected and kept), artificial food sources began to be available to storks; during only a single decade, the number of pairs nesting in the vicinity of landfills in one of the Spanish provinces represented 17 to 45% of all breeding birds (Rey 2009). In Spain and Algeria, consequences of artificial food sources were found to involve both an increase in clutch size and in hatchability (Djerdali et al. 2016a, 2008b; Tortosa et al. 2002). The lack of impact of the proximity of landfills on breeding efficiency in our study may be caused by the age of the relevant individuals. White storks observed foraging on landfills in Poland were mostly immature, in their second year of life (Bialas et al., in prep.). Juveniles show a higher level of plasticity than adults (Heinrich 1995; Greenberg 2003; Biondi et al. 2010); hence, they are more prone to using new food sources, but also, they have lower levels of breeding success than older birds (Sergio et al. 2011).

New feeding opportunities for the Central-Eastern European population of the white stork may have tremendous consequences for population trends. We already know that this population is using landfills in non-breeding grounds (Ciach and Kruszyk 2010). This behaviour is known to affect migration. In Western Europe, population changes in migration routes caused by anthropogenic sources of food are enormous, shortening migration and sometimes eliminating it (Gilbert et al. 2016). The collapse of the population in Western Europe may have been caused by reduced rainfall in its wintering grounds in Africa (Kanyamibwa et al. 1990, 2007; Bairlein 1991). Population trends may be regulated not by breeding effect but by a low recruitment rate due to a high rate of mortality on the wintering grounds (Kanyamibwa et al. 2007). Hence, the capacity to survive winter, which comes with the availability of new food sources, is probably the main cause of the recent Western European population rebuilt after the collapse. With this in mind, we can expect a similar process to occur in the Central-Eastern European population of the white stork.

## Conclusions

Our results clearly show that the most important factors in the productivity and nest-site selection of the white stork are land use and nesting structure. We have also observed the growing importance of artificial food sources at landfills, something that should be thoroughly studied in the future. White stork behavioural plasticity was a key factor in the success of this species in the past, and as the species faces changes in land use and climate, it will probably constitute a key factor in its survival in future.

## Electronic supplementary material

ESM 1 (DOC 108 kb)
