# Supplementary material for: Determination of nest occupation and breeding effect of the white stork by human-mediated landscape in Western Poland
Source: Environ Sci Pollut Res Int. 2019 Dec 11;27(4):4148–58. doi: 10.1007/s11356-019-06639-0 (PMC7024061; doi:10.1007/s11356-019-06639-0)
Supplement: Supplementary file 1 — (DOC 108 kb) [file 11356_2019_6639_MOESM1_ESM.doc]

**Electronic Supplementary Materials**

**Determination of nest occupation and breeding effect of the white stork by human-**mediated landscape in Western Poland

Joanna T. Bialas*, Łukasz Dylewski, Marcin Tobolka

Institute of Zoology, Poznań University of Life Sciences, Wojska Polskiego 71C, 60-625 Poznań, Poland

*Corresponding author. E-mail address: jtwozna@gmail.com (J.T. Bialas)

Fig S1. The relationship between the probability of nest occupation and land cover types (A, B)


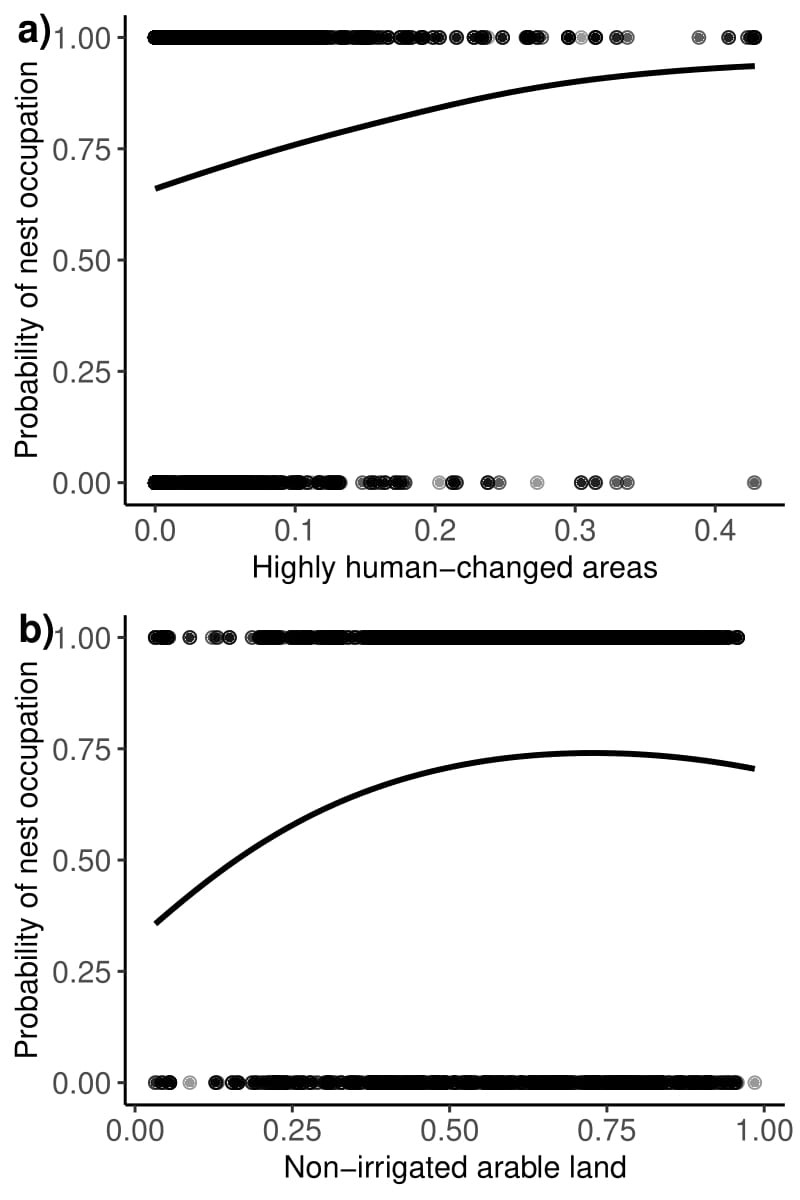


Table S1 Result of the model selection procedure on probability of nest occupation.

| **Model** | **df** | **logLink** | **AICc** | **delta** | **weight** |
| --- | --- | --- | --- | --- | --- |
| **nest_str** | 7 | -1909.907 | 3833.8 | 64.83 | 0.000 |
| **nest_str + dist_land** | 8 | -1908.255 | 3832.5 | 63.53 | 0.000 |
| **nest_str + dist_land + land +nest_str * dist_land** | 13 | -1902.741 | 3831.6 | 62.55 | 0.000 |
| **nest_str+dist_land+land+nest_str*dist_land+as.factor(year)*dist_land** | 33 | -1868.897 | 3804.3 | 35.25 | 0.000 |
| **nest_str+dist_land+human+land+I(arable^2)+arable+agri_land+meadow** | 14 | -1895.527 | 3819.1 | 50.13 | 0.000 |
| **nest_str+dist_land+human+land+I(arable^2)+arable+agri_land+meadow+as.factor(year)*dist_land** | 34 | -1861.396 | 3791.3 | 22.28 | 0.000 |
| **as.factor(year):dist_land+nest_str+dist_land+human+I(arable^2)+arable+agri_land+meadow+nest_str*dist_land** | 27 | -1865.053 | 3784.4 | 15.41 | 0.000 |
| **as.factor(year):dist_land+nest_str+dist_land+human+I(arable^2)+arable+agri_land+meadow+nest_str*dist_land+nest_str*human+nest_str*arable+nest_str*agri_land+nest_str*meadow** | 43 | -1842.704 | 3772.2 | 3.19 | 0.169 |
| **as.factor(year):dist_land+nest_str+dist_land+human+land+I(arable^2)+arable+agri_land+meadow+nest_str*dist_land+nest_str*human+nest_str*land+nest_str*arable+nest_str*agri_land+nest_str*meadow** | 48 | -1836.013 | 3769.0 | 0.00 | 0.831 |

Table S2 Result of the model selection procedure on breeding effect.

| **Model** | **df** | **logLink** | **AICc** | **delta** | **weight** |
| --- | --- | --- | --- | --- | --- |
| **nest_str** | 8 | -4575.979 | 9168.0 | 27.90 | 0.000 |
| **nest_str + dist_land** | 9 | -4575.978 | 9170.0 | 29.91 | 0.000 |
| **nest_str + dist_land + land +nest_str * dist_land** | 14 | -4572.990 | 9174.1 | 34.02 | 0.000 |
| **nest_str+dist_land+land+nest_str*dist_land+as.factor(year)*dist_land** | 34 | -4540.515 | 9149.9 | 9.75 | 0.007 |
| **nest_str+dist_land+human+land+I(arable^2)+arable+agri_land+meadow** | 15 | -4571.649 | 9173.5 | 33.36 | 0.000 |
| **nest_str+dist_land+human+land+I(arable^2)+arable+agri_land+meadow+as.factor(year)*dist_land** | 35 | -4538.839 | 9148.6 | 8.45 | 0.014 |
| **as.factor(year):dist_land+nest_str+dist_land+human+I(arable^2)+arable+agri_land+meadow+nest_str*dist_land** | **28** | **-4541.772** | **9140.1** | **0.00** | **0.977** |
| **as.factor(year):dist_land+nest_str+dist_land+human+I(arable^2)+arable+agri_land+meadow+nest_str*dist_land+nest_str*human+nest_str*arable+nest_str*agri_land+nest_str*meadow** | 44 | -4532.361 | 9154.1 | 13.99 | 0.001 |
| **as.factor(year):dist_land+nest_str+dist_land+human+land+I(arable^2)+arable+agri_land+meadow+nest_str*dist_land+nest_str*human+nest_str*land+nest_str*arable+nest_str*agri_land+nest_str*meadow** | 49 | -4531.396 | 9162.5 | 22.40 | 0.000 |
